# VE-Cadherin and Vesicles Differentially Regulate Lymphatic Vascular Permeability to Solutes of Various Sizes

**DOI:** 10.3389/fphys.2021.687563

**Published:** 2021-09-21

**Authors:** Melanie Jannaway, Joshua P. Scallan

**Affiliations:** Molecular Pharmacology and Physiology, Morsani College of Medicine, University of South Florida, Tampa, FL, United States

**Keywords:** barrier function, molecular weight, solute flux, endothelium, lymphatic vasculature

## Abstract

Lymphatic vascular permeability prevents lymph leakage that is associated with lymphedema, lymphatic malformations, obesity, and inflammation. However, the molecular control of lymphatic permeability remains poorly understood. Recent studies have suggested that adherens junctions and vesicle transport may be involved in regulating lymphatic vessel permeability. To determine the contribution of each transport pathway, we utilized an *ex vivo* permeability assay to directly measure the solute flux of various molecular weight solutes across a range of pressures in intact murine collecting lymphatic vessels. Pharmacological and biological tools were used to probe the relative contributions of vesicles and junction proteins in the lymphatic vasculature. We show that the permeability of collecting lymphatic vessels is inversely related to the solute molecular weight. Further, our data reveal that vesicles selectively transport BSA, as an inhibitor of vesicle formation significantly decreased the permeability to BSA (∼60% decrease, *n* = 8, *P* = 0.02), but not to 3 kDa dextran (*n* = 7, *P* = 0.41), α-lactalbumin (*n* = 5, *P* = 0.26) or 70 kDa dextran (*n* = 8, *P* = 0.13). In contrast, disruption of VE-cadherin binding with a function blocking antibody significantly increased lymphatic vessel permeability to both 3 kDa dextran (5.7-fold increase, *n* = 5, *P* < 0.0001) and BSA (5.8-fold increase, *n* = 5, *P* < 0.0001). Thus, in the lymphatic vasculature, adherens junctions did not exhibit selectivity for any of the solutes tested here, whereas vesicles specifically transport BSA. Overall, the findings suggest that disease states that disrupt VE-cadherin localization or expression will cause significant leakage of solutes and fluid from the lymphatic vasculature.

## Introduction

The lymphatic vasculature constantly removes the capillary filtrate from the tissues and returns this protein-rich fluid to the blood circulation, thereby preventing fluid accumulation in the form of edema. Therefore, the permeability of the lymphatic vasculature must be tightly regulated. Recent studies have demonstrated that the permeability of the lymphatic vasculature extends beyond mere fluid balance, with major roles in several vascular diseases.

Dysregulated lymphatic vascular permeability is most easily observed as lymph leakage. Leaky lymphatic vessels have been proposed to cause adult-onset obesity (Harvey et al., 2005; Sawane et al., 2013) and are a consequence of chronic type 2 diabetes (Scallan et al., 2015) and hypercholesterolemia (Lim et al., 2009). Lymphatic vessel leakage is also a hallmark of lymphatic malformations, which are aberrant growths of the lymphatic vasculature caused by somatic or congenital gene mutations (Brouillard et al., 2014; Ozeki and Fukao, 2019). Lymphatic leakage in lymphatic malformations manifest as chylothorax, pleural effusion, and/or chylous ascites, which can be life threatening conditions. Gene mutations that disrupt lymphatic vascular permeability cause congenital lymphedema (Lapinski et al., 2012; Sabine et al., 2015), a disease characterized by incurable tissue swelling. Similarly, cancer-related lymphedema occurs in response to a Th2 inflammation that drives the accumulation of fatty fibrotic tissue and also exhibits leaky lymphatics (Avraham et al., 2013; Ogata et al., 2016; Kataru et al., 2019). Finally, dysregulated lymphatic vascular permeability has been associated with impaired immune responses as a consequence of prior infection (Fonseca et al., 2015).

However, it remains unknown how lymphatic permeability is regulated, both at functional and molecular levels. In the blood vasculature, the key junction protein that regulates vascular permeability is the adherens junction protein, VE-cadherin (Dejana et al., 2008). Reduced expression, internalization, or disorganization of VE-cadherin at cell-cell junctions is usually associated with disrupted endothelial barrier function (Dejana et al., 2008; Orsenigo et al., 2012; Grimsley-Myers et al., 2020). In the lymphatic vasculature, VE-cadherin was originally identified to contribute to discontinuous “button” junctions in the lymphatic capillaries and to continuous “zipper” junctions in the downstream collecting lymphatic vessels (Baluk et al., 2007), but this detailed morphological analysis failed to provide functional information about VE-cadherin in the lymphatic vasculature. Most recently, we generated a conditional knockout allele targeting *Cdh5*, the gene encoding VE-cadherin, to reveal that lymphatic-specific deletion of VE-cadherin causes chylous ascites (Yang et al., 2019), suggesting that VE-cadherin may regulate lymphatic permeability, although this was not investigated in detail. Intriguingly, genetic deletion of VE-cadherin from all endothelial cells *in vivo* led to increased permeability in the heart and lung vasculatures, but not in the skin or brain vasculatures, indicating that VE-cadherin may not be absolutely required for maintaining a low vascular permeability at those sites (Frye et al., 2015).

As an alternative mechanism governing lymphatic vascular permeability, vesicles in lymphatic endothelium have been proposed to regulate solute transport into the lymphatic vasculature at the level of the lymphatic capillaries (Triacca et al., 2017). Impaired vesicle transport inhibited the flux of albumin and other large solutes across lymphatic endothelium. However, this study was conducted almost entirely using cultured lymphatic endothelial cells (LEC), and it remains unclear whether an LEC monolayer is representative of lymphatic capillary or collecting lymphatic endothelium, or whether vesicular transport occurs in intact lymphatic vessels.

To begin to address how lymphatic vessels regulate their permeability, we designed the current study to test two hypotheses: (1) lymphatic vascular permeability is significantly different for solutes of varying molecular weight, and (2) lymphatic vascular permeability to albumin, but not other solutes, is dominated by vesicle-mediated transport. Here, we used photometry to quantify the solute flux across single collecting lymphatic vessels excised from the mouse. We improved this assay by adding a dual photometer system to enable the measurement of solute flux to two fluorescent solutes simultaneously, and by adding a fine pressure control system to enable repeated measures of solute flux over a range of physiological pressures in the same vessel. Pharmacologic inhibitors of vesicle transport and biologic inhibitors of VE-cadherin were used to determine the relative contribution of each transport pathway to lymphatic solute permeability (P_*s*_, cm s^–1^). Finally, we used several fluorescent solutes of various molecular weights to test whether P_*s*_ was affected by solute size. Our results show that lymphatic P_s_ is inversely related to molecular weight and directly related to pressure. However, vesicle inhibition specifically targeted albumin transport, whereas all solutes were sensitive to disrupted adherens junctions.

## Materials and Methods

### Mice

Animal protocols were reviewed and approved by the Institutional Animal Care and Use Committee (IACUC) at the University of South Florida and conformed to the National Institutes of Health *Guide for the Care and Use of Laboratory Animals.* All animals in this study were wild-type (WT) mice purchased from The Jackson Laboratory (Bar Harbor, ME) on a C57BL/6J background and were used between 6 and 8 weeks of age (∼25 g weight). Both male and females were used for experiments and had *ad libitum* access to food and water.

### Solutions and Chemicals

Krebs buffer was prepared fresh weekly and contained (in mmol): 141.4 NaCl, 4.7 KCl, 2 CaCl_2_.2H_2_O, 1.2 MgSO_4_, 1.2 NaH_2_PO_4_.H_2_O, 3 NaHCO_3_, 1.5 Na-HEPES, 5 d-glucose, and 1 g L^–1^ bovine serum albumin (BSA, Thermo Scientific, #J10856-22) added to ddH_2_O. The pH was adjusted to 7.4 ± 0.05.

Dynasore (Sigma), an inhibitor of dynamin-dependent vesicle transport, was dissolved in dimethyl sulfoxide (DMSO) to a stock concentration of 100 mM and stored at –20°C. On the day of use, an aliquot of this stock solution was thawed and diluted to 100 μM in Krebs buffer. A vehicle control was made by diluting the same volume of DMSO in Krebs buffer. Vessels were superfused continuously with the vehicle control followed by the Krebs buffer containing dynasore for paired measurements.

A monoclonal function blocking antibody raised against the extracellular domain of VE-cadherin (clone BV13) (Corada et al., 1999) or an antibody control (rat IgG) were diluted in Krebs buffer to a final concentration of 100 μg/mL and used to fill the cannulation pipettes for perfusion through the vessel lumen for the duration of the experiment. Thus, each vessel was exposed to either the antibody control or the function blocking antibody, so these experiments were unpaired.

### Fluorescent Tracers

BSA (Thermo Scientific, #J10856-22) and α-lactalbumin (Sigma, L6010) were conjugated to AF488 or AF594 (Invitrogen, #A20000 or #A20104) according to the manufacturer protocol. After proteins were conjugated, the excess free dye and DMSO were removed from the fluorescent tracer by repeated washing using centrifugal molecular weight cutoff filters (Millipore Sigma, Amicon Ultra-15, #UFC903024). The filtrate fluorescence was monitored on an inverted fluorescence microscope. The final protein concentration was measured using a BCA assay (Pierce) and the proteins were aliquoted and stored at –80°C. On the day of use, an aliquot was thawed, and the protein was added to 2 mL of Krebs buffer to a final concentration of 150 nmol mL^–1^ for each fluorescent tracer to maintain similar brightness.

Other fluorescent tracers that were used included: sodium fluorescein (NaFl), 3 kDa dextran conjugated to AF488 or Texas Red (TR), and 70 kDa dextran conjugated to AF488 or TR. All fluorescent tracers were used at a final concentration of 150 nmol mL^–1^.

### Surgical Procedure

Mice were anesthetized by an injection of ketamine (100 mg kg^–1^, i.p.) followed by an injection of thiobutabarbital (Inactin, 100 mg kg^–1^, i.p.). Once in a surgical plane of anesthesia, confirmed by lack of response to toe-pinch, the abdomen was shaved, and mice were placed supine on an acrylic board next to a pedestal made from Sylgard 184 (Ellsworth Adhesives). A 1–2 cm midline incision was made through which the small intestine and associated mesentery were exteriorized. The intestine was gently manipulated so that the mesentery laid flat on the Sylgard pedestal and the tissues were kept moist with periodic application of Krebs buffer. Collecting lymphatic vessels were identified as small vessels that ran parallel to the feeding artery/vein pairs that were frequently white in appearance, lacked red blood cells, and contained bicuspid valves. A pair of fine Graefe forceps (Fine Science Tools (FST), #11152-10) and Vannas-Tübingen spring scissors (FST, #15003-08) were used to excise several (∼3–4) collecting lymphatic vessels and their associated adipose and connective tissues from the mouse mesentery that were placed immediately in Krebs buffer in a petri dish. Once vessel harvesting was complete, mice were killed by an overdose of anesthetic followed by cervical dislocation in agreement with the IACUC protocol.

### Lymphatic Vessel Dissection and Cleaning

Individual collecting lymphatic vessels, ensheathed in connective tissue and adipocytes, were transferred to an acrylic dissection chamber recessed into a hole in the table. The contents of the dissection chamber were visualized with a stereo dissection microscope with Greenough optics (Zeiss Stemi 508) and an LED light source. The bottom of the dissection chamber had a layer of Sylgard 170 (Ellsworth Adhesives). Prior to transferring the vessels, the chamber was filled with Krebs buffer and two stainless steel pins (cut from 40 μm wire; Danish Myo Technology, #400447) were inserted into the Sylgard. The collecting lymphatic vessel was then pinned at each end to lie flat against the Sylgard bottom to approximately the same length as *in vivo*. Curved fenestrated ultra-fine Moria forceps (FST, #11399-87) were further hand sharpened and used to gently tease the connective tissue apart to facilitate location of the collecting lymphatic vessel within the adipose tissue. Then ultra-fine spring scissors (FST, #15396-00 or World Precision Instruments (WPI), #14124) were hand sharpened and used to carefully cut away the adipose and connective tissues from the lymphatic vessel, taking care to avoid stretching or cutting the vessel. Once most of the adipose tissue was removed from the vessel, each end of the vessel was cut to free it from the steel pins. A glass Pasteur transfer pipette with a fire polished tip was siliconized (ThermoFisher, #TS42800) and used to transfer the cleaned collecting lymphatic vessel to the cannulation chamber.

### Glass Pipette Fabrication and Vessel Cannulation

Glass pipettes used for vessel cannulation were pulled from capillary tubing on a vertical pipette puller (Narishige, #PC-10). The pulled glass pipettes were then broken at a diameter of 60 μm, fire polished, and bent to a 30° angle using a platinum filament with a glass bead on a microforge (Narishige, #MF-900). Pipettes were then cleaned by vacuum aspirating ddH_2_O, followed by pure acetone until dry and stored in a dust free container.

Glass pipettes were positioned within a custom machined acrylic cannulation chamber that was fixed to a transferrable stage made from an aluminum breadboard (Thorlabs) by custom machined pipette holders that enabled separate pressure control of each lumen of the theta pipette. The two pipette holders were mounted on brass rods (WPI, #5444) and were positioned at each end of the cannulation chamber by manual 3-axis manipulators (Scientifica, #LBM-7) mounted to the breadboard. Glass pipettes were backfilled with Krebs buffer with or without fluorescent tracers through low protein binding syringe filters (Millipore Sigma, #SLHVR04NK) before being lowered into the cannulation chamber that was also filled with Krebs buffer.

Collecting lymphatic vessels were cannulated on the two glass pipettes using fine, hand sharpened Dumont #5/45 forceps (FST, #11251-35). The inflow side of the collecting lymphatic vessel was identified and cannulated on a theta pipette that had two parallel lumens separated by a septum (WPI, #TST150-6). Once this side was cannulated, it was secured by tying a single knot of unbraided 4-0 suture (Roboz, #SUT-15-2) over the vessel. The downstream outflow side of the vessel was then cannulated on a single lumen glass pipette (WPI, #TW150-6) and secured with another knot of suture. Once the vessel was cannulated, the entire cannulation stage was transferred to an inverted fluorescence microscope (Zeiss Observer Z1). The water-jacketed acrylic cannulation chamber was connected to a heat exchanger pump (Lauda Alpha A6, #LCB4733-16-0002) to warm the Krebs buffer and vessel to 37°C over a ∼1-h period.

### Dual Photometer

To enable us to determine the permeability of collecting lymphatic vessels to two different solutes simultaneously for the first time, we modified the photometer by adding two photomultiplier tubes (PMT) at right angles (Horiba/PTI, D-104). The inverted microscope was capable of fluorescence imaging and was outfitted with an LED light source (Excelitas Technologies, X-cite Xylis) and a sensitive 16-bit CMOS camera (Hamamatsu Flash 4.0). A dichroic filter (Chroma, T565lpxr) was mounted in a Nikon filter cube inside the dual photometer and separated incoming fluorescence emission into “green” and “red” wavelengths that were directed to each PMT. Immediately before each PMT, an emission filter was used to restrict incoming light to the range of wavelengths for that particular PMT (Chroma ET525/50m and ET645/75m). To prevent overexciting the PMTs with excessive light, a 0.1% transmission neutral density filter (Chroma, #UVND 3.0) was inserted into the light path prior to each measurement to limit the intensity of the excitation beam. A dual bandpass filter (Chroma, 59022) was installed in the inverted microscope to simultaneously excite and emit “green” and “red” wavelengths that corresponded to the fluorescence spectra of Alexa Fluor (AF) 488 and AF594.

### Pressure Control System

In previous studies we had used a manual water manometer to control the pressures in each lumen, but this was problematic because pressures changed over time due to forward flow changing the fluid levels of the reservoirs. To overcome this problem, we installed a new automated pressure control system to finely control the pressure in each of the three glass pipette lumens. The vessel was attached to the pressure system using PE-190 tubing. Low pressure transducers (CyQ model 104) were attached in-line with the tubing with 3-way valves (Hamilton, HV3-3) to monitor the vessel pressure. The pressures from the transducers were displayed on low voltage amplifiers (Omega Engineering, DP41-E-A) with analog BNC outputs fed into a data acquisition device (National Instruments, USB-6341). The photometer BNC outputs were also fed into the same data acquisition device. Custom written LabView software was used to display and record the pressures and photometer voltages at 30 Hz on a digital chart recorder. Additionally, the LabView software controlled the pressure by sending pulses to a pressure controller (Elvesys, Elveflow OB-1) attached to fluid reservoirs in-line with the vessel tubing.

### Protocols to Quantify Solute Flux

To assess the solute permeability of a collecting lymphatic vessel, solute flux was measured using the photometer at several pressures over a physiological pressure range (5–20 cmH_2_O). The luminal hydrostatic pressure of mouse lymphatic vessels has to date not been measured. Thus, our pressure range was drawn from previous studies including that by Bouta et al. (2014), that indirectly measured maximal lymphatic pumping pressure in mouse leg lymphatics using a cuff, showing it to be 11 cmH_2_O, and a report by Zweifach and Prather (1975) that found the pressure in rat collecting lymphatics to range from 12 to 18 cmH_2_O. Using the dual photometer system, the solute flux of two solutes could be quantified by the photometer simultaneously. At each pressure, after the solute flux trace had been acquired, a calibrated brightfield image of the vessel was obtained in Zen Blue software using the CMOS camera. Using the distance function in the Zen software, the diameter of the vessel was obtained and recorded for later analysis. If a pharmacologic treatment was used, then the solute flux at each pressure was measured in the presence of a vehicle control, and this was repeated in the presence of the pharmacologic inhibitor. The biological inhibitor used in this study was perfused through the vessel lumen, so each vessel was exposed to either a control or function blocking antibody for unpaired measurements.

### Determination of Solute Permeability From Solute Flux

**FIGURE 1:**
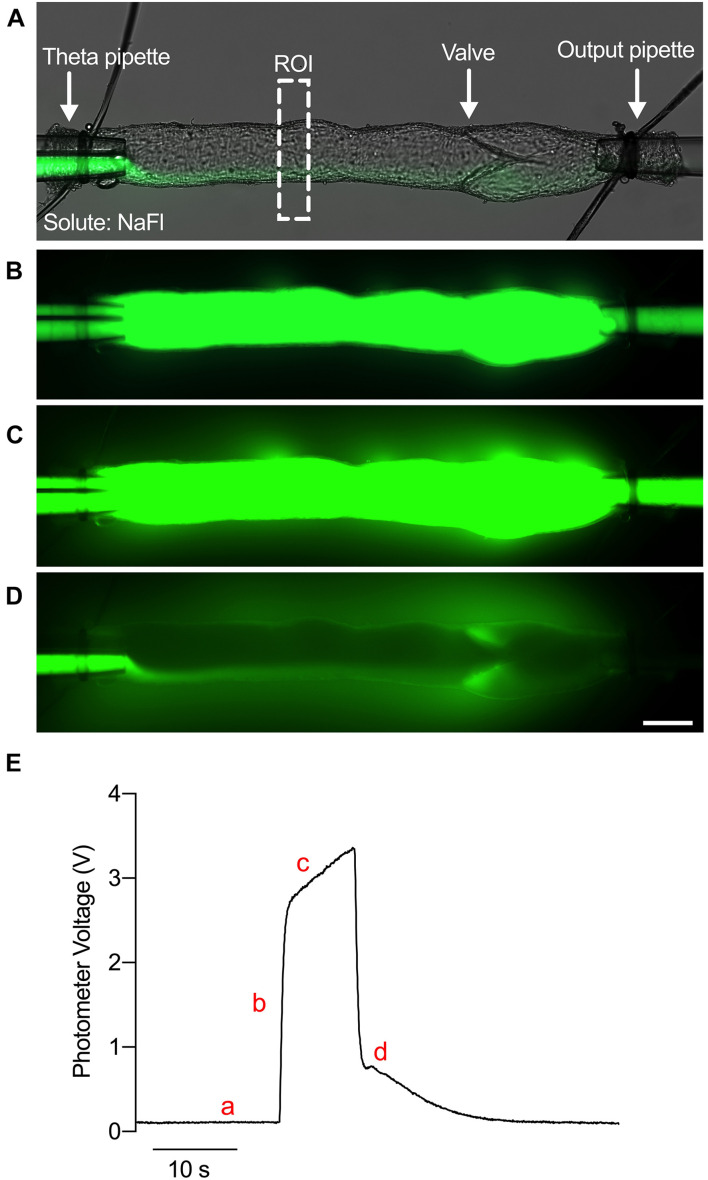
*Ex vivo* assay to determine the permeability of collecting lymphatic vessels. **(A)** A brightfield image of an isolated, cannulated collecting lymphatic vessel from mouse mesentery with an overlaid fluorescence image of NaFl. ROI, region of interest. **(B)** Fluorescence image of NaFl perfusion through the lumen. **(C)** Fluorescence image of NaFl perfusion several seconds after the image in panel **(B)** depicting leakage of NaFl into the bath solution. **(D)** Fluorescence image of the same vessel immediately after washing out the NaFl with Krebs buffer. Extravascular NaFl can be observed. **(E)** A raw trace of the photometer voltage elicited by photons in the ROI during perfusion of Krebs buffer (a), immediately after perfusion of NaFl (b), several seconds after perfusion of NaFl (c), and immediately after washing out the NaFl with Krebs buffer (d). Scale bar is 100 μm.

The P_s_ at each pressure is determined from the solute flux measured during the experiment as previously described (Huxley et al., 1987; Scallan and Huxley, 2010). To directly measure solute flux, the fluorescence intensity of a rectangular region of interest (ROI) encompassing the vessel lumen and the bath solution on either side of the vessel was continuously quantified with the dual photometer (Figure 1A). An example trace of voltage over time is shown in Figure 1E. The vessel was first perfused with the washout Krebs buffer, and a minimal amount of probe solution was allowed to perfuse the vessel to prevent mixing of the washout and probe solutions (Figure 1A). Thus, a baseline level of voltage was observed (Figure 1Ea). Upon switching the input pressures, the vessel lumen was filled completely with the fluorescent probes causing a step increase in voltage (Figures 1B,Eb). As solute crossed the lymphatic endothelial barrier and accumulated in the bath solution over time (Figure 1C), a linear increase in the photometer voltage was generated (Figure 1Ec). When the input pressures were switched again, the fluorescent probes were washed out to return the voltage to baseline (Figures 1D,Ed). Thus, the initial stepwise increase in fluorescence intensity (I_*o*_) fills only the vessel lumen with fluorescent tracer and is proportional to the number of molecules of solute (mol). Afterward, the gradual linear increase in fluorescence intensity (dI_*f*_/dt) is the solute flux across the vessel wall (J_s_, mmol s^–1^). Finally, the volume to surface area ratio is equivalent to the diameter of the vessel divided by 4 (D/4). By rearranging Fick’s First Law, we obtain Eq. 1:


(1) P=s(1/I)o(dI/fdt)(V/S)

which allows one to calculate the solute permeability (P_s_, cm s^–1^) of a given vessel. Each solute flux trace measured by the photometer and recorded by LabView was plotted in Prism GraphPad software to perform linear regression to obtain the slope of the solute flux portion of the trace (dI_*f*_/dt). Prism software was also used to obtain the height of the step increase in fluorescence intensity (I_*o*_). The diameter of the vessel was obtained from a brightfield image of the vessel at that particular pressure acquired by the CMOS camera using the distance function in Zen Blue software.

### Immunofluorescence of Isolated Collecting Lymphatic Vessels

Vessels were isolated, cannulated and pressurized at 10 cmH_2_O, as described above. For imaging VE-cadherin, vessels were then treated with either vehicle (DMSO) or dynasore (100 μM) for 30 min, or with IgG isotype control or BV13 (100 μg) for 4 h and subsequently fixed with 2% PFA while remaining pressurized for 5–10 min. The vessels were then uncannulated and transferred to a siliconized petri dish containing 2% PFA and fixed overnight at 4°C. The next day, vessels were washed with PBS three times, 10 min each, permeabilized with PBS + 0.3% triton-X (PBST) for 1 h, and then blocked with 3% donkey serum for 2 h, all at 4°C. Vessels were then incubated with primary antibodies for PROX1 (1:1000; Abcam cat#: ab101851) and VE-cadherin (1:500; BD Biosciences cat#: 550548) overnight at 4°C. Following five washes, 10 min each with PBST, vessels were incubated at room temperature with the appropriate secondary antibodies (donkey anti-rabbit AF488, donkey anti-rat AF594) for 2 h. Vessels were washed again in PBST, then incubated with DAPI at room temperature for 5 min. After a final wash in PBS, the vessels were mounted on glass slides with spacers (Invitrogen, cat#: S24735) and imaged on a Leica TCS SP8 confocal microscope equipped with a 40× water immersion objective.

For imaging BSA and 70 kDa contained within vesicles, vessels were isolated, cannulated and pressurized at 10 cmH_2_O, then pre-treated with either vehicle (DMSO) or dynasore (100 μM) for 30 min, before the lumen of the vessel was filled with BSA-AF488 and 70 kDa dextran-TR for 1 h. The fluorescently labeled solutes were then washed out of the lumen by switching the pressures controlling the theta pipette, and the vessels lightly fixed with 2% PFA for 3 min. The vessels were then uncannulated and immediately mounted with ProLong Diamond Antifade Mountant with DAPI (ThermoFisher cat# P36966), before being immediately imaged on a Leica TCS SP8 confocal microscope equipped with a 40× water immersion objective.

### Statistics and Data Analysis

On all graphs, “*n*” refers to a single vessel from a single mouse, so that each *n* represents a different mouse. Prism software (v8, Graphpad Software, CA) was used for statistical analysis. All data are presented as means ± SD. To compare differences between different pressures for a single solute, one-way ANOVA was performed with a Dunnett’s *post hoc* test for comparison to the lowest pressure. To compare the differences between treatment groups or solutes at multiple pressures, two-way ANOVA was performed with the Holm-Sidak *post hoc* test for multiple comparisons. Images were obtained as .czi files from the Zen Blue software that were converted to heat maps using FIJI (NIH Image J) software. Confocal images of vessels stained for PROX1 and VE-cadherin were also analyzed with FIJI software to estimate the mean pixel intensity.

## Results

### Lymphatic Vessel Permeability Is Dependent on Solute Size and Pressure

Collecting lymphatic vessels have been described as impermeable structures both historically (Mayerson, 1963) and recently (Zhang et al., 2020). However, over the past decade, our studies have demonstrated that collecting lymphatic vessels *in vivo* and *ex vivo* are not only permeable, with a permeability to albumin similar to that of post-capillary venules ∼3 × 10^–7^ cm s^–1^, but also leak solute from the lumen into the surrounding tissue due to their high hydrostatic pressure (Scallan and Huxley, 2010; Scallan et al., 2015). Work from other groups have supported the finding that collecting lymphatic vessels *in vivo* are permeable (Fonseca et al., 2015; Kuan et al., 2015). Information gained from recent *ex vivo* studies was limited because the permeability of collecting lymphatic vessels was only assessed at a single pressure and to a single solute, albumin, that has a high molecular weight (Scallan et al., 2015; Ivanov et al., 2016). Thus, it is currently unknown whether the permeability of lymphatic vessels is sensitive to solute size or pressure.

**FIGURE 2:**
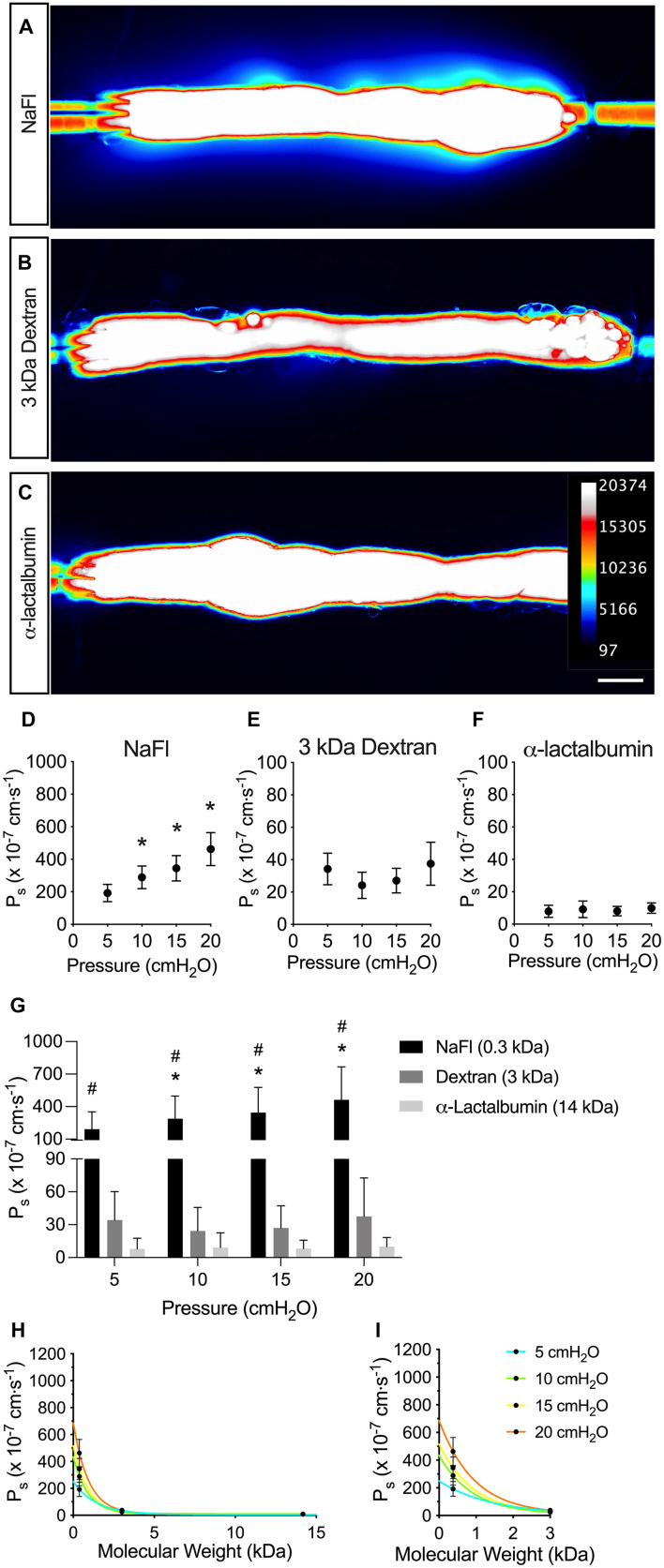
Solute permeability is inversely related to solute size. Solute flux was measured to three different solutes across a range of physiologically relevant sizes and solute permeability (P_s_) quantified. **(A–C)** Visualized leakage of each indicated solute from collecting lymphatic vessels. Fluorescence intensity of each solute was captured with a CMOS camera and the intensity converted to a heat map (see inset for scale). **(D–F)** P_s_ to each indicated solute plotted against pressure to show mean values and convective flux (NaFl, *n* = 9; 3 kDa dextran and α-lactalbumin, *n* = 7). **(G)** A summary of the solute fluxes from panel **D–F** plotted on the same graph for comparison. Significant differences in P_s_ were detected between all three solutes with two-way ANOVA. **(H)** P_s_ is plotted against molecular weight of each solute. Each curve represents a different pressure (NaFl, *n* = 9; 3 kDa dextran and α-lactalbumin, *n* = 7). **(I)** A magnified view of the graph in panel **(H)** at molecular weights of 3 kDa and below. The P_s_ of the low molecular weight solute, NaFl (0.3 kDa), is especially pressure-dependent. All graphs show mean ± SD and each “*n”* refers to a different mouse. *, *P* < 0.05 when NaFl is compared to 3,000 Da dextran. #, *P* < 0.05 when NaFl is compared to α-lactalbumin. Scale bar is 100 μm.

To test the effect of solute size on collecting lymphatic vessel permeability, we used the assay shown in Figure 1 across a range of pressures from 5 to 20 cmH_2_O and across a range of solute sizes from 0.376 to 14 kDa. Collecting lymphatic vessels were visibly permeable to the smallest solute tested, NaFl (*MW* = 376 Da) (Figure 2A). Similarly, the flux of 3 kDa dextran out of the vessels could be viewed by eye under the microscope (Figure 2B). However, flux of the next larger protein α-lactalbumin (*MW* = 14,178 Da) was no longer visible by eye and could only be detected by the photometer (Figure 2C).

When P_s_ was determined from the flux of NaFl and plotted in Figure 2D, it increased linearly with pressure, indicating that NaFl permeability was pressure-dependent. The mean P_s_ to NaFl was 192 ± 156, 289 ± 209, 344 ± 234, and 462 ± 304 × 10^–7^ cm s^–1^ at pressures of 5, 10, 15, and 20 cmH_2_O, respectively (*n* = 9). The P_s_ to 3 kDa dextran was significantly decreased compared to NaFl (*P* < 0.0001) (Figures 2E,G). The mean P_s_ to 3 kDa dextran was 34.2 ± 25.9, 24.2 ± 21.6, 27.1 ± 20.2, and 37.5 ± 35.1 × 10^–7^ cm s^–1^ at each pressure (*n* = 7). The P_s_ to α-lactalbumin did not appear to be pressure dependent (Figure 2F) and P_s_ to α-lactalbumin was significantly decreased compared to NaFl (*P* < 0.0001) and 3 kDa dextran (*P* < 0.0001) (Figure 2G). The mean P_s_ to α-lactalbumin was 7.88 ± 9.83, 9.14 ± 13.5, 8.05 ± 7.78, and 9.93 ± 8.41 × 10^–7^ cm s^–1^ at pressures of 5, 10, 15, and 20 cmH_2_O, respectively (*n* = 7).

To determine the relationship between solute size and collecting lymphatic vessel permeability to the solute, the P_s_ of all solutes were plotted against molecular weight (Figure 2H). In general, the P_s_ decreased exponentially as molecular weight increased. Additionally, the effect of pressure on P_s_ was more prominent for the solutes with a molecular weight of 3 kDa and below (Figure 2I). There was a clear increase in P_s_ to NaFl with increasing pressure, but this effect was diminished for 3 kDa dextran and α-lactalbumin.

### Vesicular Transport Contribution to Lymphatic Vessel Permeability

Studies in the blood vasculature have long demonstrated a role for vesicles in transporting albumin across the endothelium (Ghitescu et al., 1986; Milici et al., 1987; Schubert et al., 2001). Other studies have shown that albumin binds receptors expressed on caveolae and vesicles (Tiruppathi et al., 1996; Vogel et al., 2001). More recently, a study of cultured LECs demonstrated that the transport of BSA and 70 kDa dextran is dependent upon vesicular transport, since the dynamin inhibitor, dynasore, significantly reduced solute uptake and release of both of these solutes (Triacca et al., 2017). However, whether vesicles transport solutes in intact lymphatic vessels remains unclear.

**FIGURE 3:**
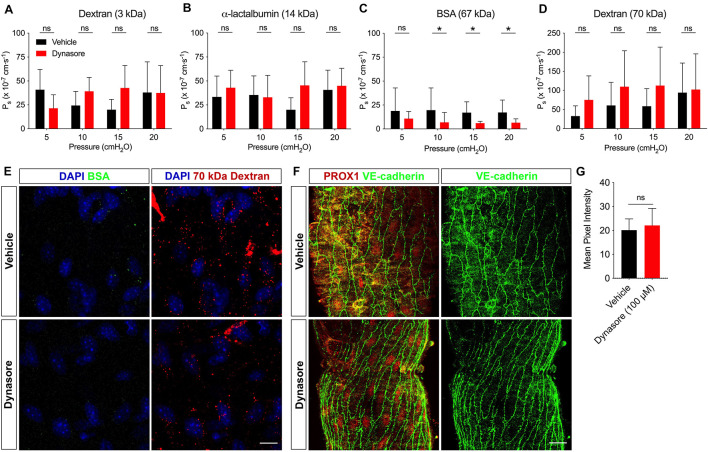
BSA utilizes vesicular transport to cross the lymphatic barrier. P_s_ of collecting lymphatic vessels to **(A)** 3 kDa dextran (*n* = 7), **(B)** α-lactalbumin (*n* = 5), **(C)** BSA (*n* = 8), or **(D)** 70 kDa dextran (*n* = 8) was determined first in the presence of vehicle (black bars), followed by dynasore (red bars), an inhibitor of vesicle formation. **(E)** Collecting lymphatic vessels treated with either vehicle of dynasore (100 μM) and imaged immediately after perfusion of BSA-AF488 (green) and 70 kDa dextran (red). Vesicles containing BSA-AF488 and 70 kDa dextran-TR are visible in the endothelial layer. **(F)** Representative images of collecting lymphatic vessels stained for PROX1 (red) and VE-cadherin (green) after treatment with either vehicle or dynasore (100 μM) and imaged by confocal microscopy. **(G)** Summary data of mean pixel intensity from *n* = 3 pairs of isolated, immunostained lymphatic vessels treated as in D (Student’s unpaired *t*-test; *P* = 0.71). All graphs show mean ± SD and each “*n*” refers to a different mouse. *, *P* < 0.05 by two-way ANOVA. Scale bar is 10 μm in panel **(E)** and 20 μm in panel **(F)**.

To address whether vesicles contributed to solute transport, the P_s_ of collecting lymphatic vessels to a range of solutes (3 kDa dextran, α-lactalbumin, BSA and 70 kDa dextran) was assessed before and after incubation with dynasore (100 μM). With the capacity to measure the solute flux of two solutes at a time, each vessel was perfused with a pair of solutes. The P_s_ to 3 kDa dextran (*n* = 7; *P* = 0.41) (Figure 3A) and α-lactalbumin (*n* = 5; *P* = 0.26) (Figure 3B) in the presence of dynasore was not significantly different from vehicle controls at any pressure. However, collecting lymphatic vessel P_s_ to BSA (Figure 3C) was significantly decreased in response to dynasore (*n* = 8; *P* = 0.02) by 42.6, 64.8, 64.7, and 65.3% at pressures of 5, 10, 15, and 20 cmH_2_O, respectively. The mean decrease in albumin transport across all pressures was 59.4%. To determine whether all large solutes were preferentially transported by vesicles, we determined the P_s_ to 70 kDa dextran, which is similar to BSA in molecular weight. In contrast to BSA, the P_s_ to 70 kDa dextran (Figure 3D) was not significantly different between dynasore and vehicle controls (*n* = 8; *P* = 0.13). Because albumin transport has been previously linked to vesicular transport, we next attempted to image whether BSA and 70 kDa dextran localized to vesicles. Confocal imaging of isolated lymphatic vessels showed that both BSA and 70 kDa dextran were localized to puncta reminiscent of vesicles. Following exposure to dynasore, the BSA-containing vesicles were largely absent, while the 70 kDa dextran vesicles remained (Figure 3E). Dynasore was previously shown to inhibit VE-cadherin internalization in blood endothelial cells (Semina et al., 2014), which would also be expected to decrease the permeability to albumin. Since maintaining VE-cadherin at the intercellular junctions would also decrease permeability to 70 kDa dextran, and our data showed that 70 kDa dextran permeability remained unchanged in the presence of dynasore (Figure 3D), it was unlikely that dynasore inhibited VE-cadherin internalization. However, to directly assess this possibility, single mesenteric collecting lymphatic vessels treated with either vehicle or dynasore, as before, were immunostained for VE-cadherin and imaged with confocal microscopy (Figure 3F). The mean pixel intensity did not significantly change following dynasore treatment (Figure 3G). Thus, these results are consistent with vesicles in intact collecting lymphatic vessels functioning in albumin specific transport out of these vessels.

### Junctional Regulation of Solute Flux

**FIGURE 4:**
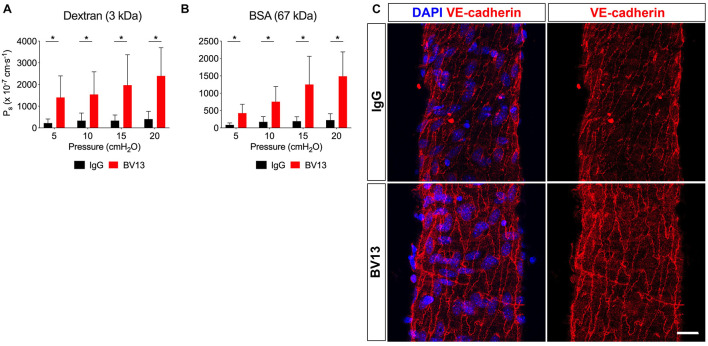
VE-cadherin regulates collecting lymphatic vessel solute permeability (P_s_). Collecting lymphatic vessels were perfused with either isotype control antibody (IgG) or a VE-cadherin function blocking antibody (clone: BV13, 100 μg/mL). **(A)** P_s_ to 3 kDa dextran in the presence of IgG antibody (black bars) or following treatment with BV13 antibody (red bars) (*n* = 5). **(B)** P_s_ to BSA in the presence of IgG (black bars) or following treatment with BV13 (red bars) (*n* = 5). **(C)** Collecting lymphatic vessels perfused with IgG or BV13 (100 μg/ml) were fixed and stained for DAPI (blue) and VE-cadherin (red). All graphs show mean ± SD and each “*n*” refers to a different mouse. *, *P* < 0.05 by two-way ANOVA. Scale bar is 20 μm.

Since vesicles did not appear to transport any of the solutes tested in this study besides albumin, we reasoned that transport through junctions is likely to be the primary route for solute flux out of the intact collecting lymphatic vessel. VE-cadherin is an adherens junction protein originally identified in blood endothelial cells to regulate vascular permeability, in addition to being known for its specific expression in all vascular endothelial cells (Lampugnani et al., 1992). In a recent study, we demonstrated that inactivation of the VE-cadherin gene leads to chylous ascites and lymph leakage 2 weeks, but not 1 week, after deletion (Yang et al., 2019), suggesting a potential role for VE-cadherin in the regulation of lymphatic vessel integrity. To determine whether adherens junctions regulate lymphatic solute permeability, we utilized a VE-cadherin function blocking antibody (clone: BV13) which physically prevents VE-cadherin molecules on adjacent cells from binding each other and elicits diffusion of VE-cadherin away from the cell-cell junctions (Corada et al., 1999). Collecting lymphatic vessels were luminally perfused with either an IgG control antibody or the BV13 function blocking antibody at a concentration of 100 μg/mL. Because the luminal perfusate could not be exchanged without recannulating the vessels, these measurements were necessarily unpaired, and lymphatic vessels were randomly separated into either the control or treatment groups. Pilot experiments were performed with BV13 to determine that the shortest incubation time that led to a significant blockade was 4 h. Notably, immunostaining of isolated collecting lymphatic vessels demonstrated that, although VE-cadherin is still primarily located at the intercellular junctions following either IgG or BV13 treatment for 4 h, it is also more diffusely spread across the endothelial cells after exposure to BV13 (Figure 4C). Luminal perfusion of BV13 for 4 h led to a significant increase in P_s_ to both 3 kDa dextran (Figure 4A; *n* = 5; *P* < 0.0001) and BSA (Figure 4B; *n* = 5; *P* < 0.0001) when compared with IgG treated vessels. The increase in P_s_ for 3 kDa dextran was 6.3, 4.6, 5.9, and 5.9-fold at pressures of 5, 10, 15, and 20 cmH_2_O, respectively, and 4.9, 4.3, 6.5, and 6.6-fold for BSA at each pressure.

## Discussion

The present study tested the hypotheses that lymphatic vascular permeability differs significantly with molecular weight of the solute, and that albumin transport is dominated by vesicular trafficking. Confirming our first hypothesis, our results with intact collecting lymphatic vessels demonstrated that solutes of smaller molecular weights have a significantly higher permeability than solutes of larger molecular weights. Pharmacologic inhibition of vesicle transport in collecting lymphatic vessels revealed that, on average across the different pressures tested, 59.4% of the total albumin flux occurred through vesicles. Interestingly, the similarly sized 70 kDa dextran did not utilize vesicular transport. In contrast, antibody blockade of VE-cadherin binding dramatically increased lymphatic vascular P_s_ to both small and large solutes.

### Solute Permeability Is Inversely Related to Solute Size

Recently, we developed an *ex vivo* assay to directly measure solute flux across collecting lymphatic vessels from mice, thus enabling the use of genetic mouse models for investigating lymphatic integrity (Scallan et al., 2015; Ivanov et al., 2016). In these and earlier studies of lymphatic vascular permeability (Scallan and Huxley, 2010; Scallan et al., 2013), we used a single solute, BSA, to probe lymphatic solute permeability at a single hydrostatic pressure. Indeed, albumin has been the main protein used to probe vascular solute permeability for the past several decades likely because it is the major contributor to plasma oncotic pressure, and because it is a macromolecule with a low basal permeability that facilitates the detection of elevated permeability. However, contemporary studies have indicated that the majority of albumin requires vesicles to be transported across the endothelium (Razani et al., 2001; Schubert et al., 2001). Thus, a potential problem with using albumin to gain information about the endothelial barrier is that it may reflect vesicular transport mechanisms instead of junctional transport mechanisms. Since junctional transport is more relevant to inflammatory disease states, this means that other solutes are likely more informative regarding vascular permeability.

One previous study attempted to relate the effective lymphatic P_s_ to solute size (Ono et al., 2005), but this study was fraught with problems. The authors reported measurements of “net flux” that are typically expressed in units of mmol s^–1^, therefore identifying solute flux as the rate of the number of molecules that leave the vessel per unit time. In this particular study, the “net flux” measurements had units of μM μm^–1^, or concentration per unit distance, so these were not likely true measures of the rate of solute flux. Another problem was that the flux of each fluorescent solute was estimated by measuring the loss of fluorescence from the vessel lumen with an insensitive 8-bit CCD camera. The insensitivity of the camera was likely responsible for the authors reporting a net flux of zero for 70 kDa dextran, even though endothelium has a non-zero permeability to many large macromolecules. Although their data suggested that lymphatic vessels may be more permeable to smaller solutes compared to larger solutes, the “net flux” of each solute could not be directly compared because the concentration gradient for each solute was different. Notably, Fick’s First Law states that solute flux changes as the concentration gradient changes. Thus, there is no information currently available in the literature regarding how solute size will affect the permeability of collecting lymphatic vessels from any species.

The data reported here are the first direct measures of collecting lymphatic vessel solute flux using solutes of various molecular weights. We demonstrate that lymphatic vascular permeability increases significantly as the molecular weight of the solute decreases. This finding suggests that the solutes tested here are moving primarily through pathways that are size-dependent, such as intercellular junctions. The P_s_ to each solute increased with pressure, demonstrating convective drag, and indicated that these solutes are moving through pathways that also conduct fluid movement, providing additional support for a junctional pathway as the major mode of solute flux in collecting lymphatic vessels. To address *how* solute is transported out of collecting lymphatic vessels, we next focused on the vesicular and adherens junction pathways.

### Vesicular Transport Specifically Regulates Albumin Transport

A recent study suggested that cultured LEC may transport large solutes such as BSA and 70 kDa dextran via dynamin-dependent intracellular vesicles (Triacca et al., 2017). In collecting lymphatic vessels, where VE-cadherin expression at “zipper” adherens junctions is continuous, vesicles could be used in the transport of large macromolecules like albumin.

Combining pharmacological ablation of vesicle formation with an *ex vivo* P_s_ assay, we show that inhibition of vesicular transport significantly reduced collecting lymphatic vessel P_s_ to BSA by 59.4%. This is strikingly similar to a previous report, which stated that ∼40% of albumin transport occurred through convection pathways and the other 60% via pressure-independent pathways, most likely comprised of vesicles (Scallan and Huxley, 2010). Furthermore, the data suggest that vesicular transport may be specific to albumin because collecting lymphatic P_s_ to 3 kDa dextran, α-lactalbumin and 70 kDa dextran were not significantly changed by dynasore treatment. This concept is supported by direct imaging of vesicles in this study showing that both BSA-AF488 and 70 kDa dextran-TR are present in vesicles in the endothelium of isolated collecting lymphatic vessels following vehicle treatment, while only 70 kDa dextran-TR-containing vesicles remain after treatment with dynasore. Endocytosis in endothelial cells can occur via a number of different mechanisms (Stan, 2006). Since 70 kDa dextran transport was unaffected by dynasore inhibition of dynamin but we can still visualize this solute inside vesicular structures, we postulate that 70 kDa dextran might be endocytosed in a dynamin independent manner. However, it was not possible to perform immunostaining for different vesicle markers at the same time as visualizing the solutes.

Our findings differ from those of Triacca et al. (2017), who suggest that both BSA and 70 kDa dextran are transported via vesicles. Possible explanations for this difference include that we used intact lymphatic vessels, whereas that study primarily used cultured lymphatic endothelial cells, and that we used vessels with a collecting lymphatic identity. Cultured LECs have an undefined identity (e.g., lymphatic capillary or collecting lymphatic), so it remains unknown how this may contribute to vesicle trafficking. Alternatively, this difference could be explained by gp60 – an albumin-binding protein shown to be important for the transcytosis of albumin (Schnitzer, 1992; Vogel et al., 2001). The presence of gp60 would offer a selective transcellular route for albumin but not 70 kDa dextran. Triacca et al. (2017) also demonstrated that, while vesicular transport occurred in both directions across cultured LECs, it was significantly higher in the basal-to-apical direction, suggesting absorption of solute. However, data here and from published studies have demonstrated that solutes move out of collecting lymphatic vessels in the apical-to-basal direction (Scallan and Huxley, 2010; Scallan et al., 2015; Ivanov et al., 2016). Therefore, this difference may be due to the possibility that cultured LECs may be functionally analogous to lymphatic capillaries that are known to absorb fluid and solute, whereas collecting lymphatic vessels have been shown to extravasate solute. Overall, our findings suggest that in collecting lymphatic vessels, the majority of BSA is transported out of the lumen via vesicles. Given that vesicle transport was specific to BSA, we next investigated adherens junctions as a route for solute flux from collecting lymphatic vessels.

### VE-Cadherin Inhibition Increases Lymphatic Vascular Permeability

Collecting lymphatic vessels express continuous VE-cadherin at the cell-cell junctions (i.e., zipper junctions) akin to arteries and veins (Baluk et al., 2007; Yang et al., 2019). Since VE-cadherin is a major regulator of blood vessel permeability (Dejana and Vestweber, 2013), it is logical that collecting lymphatic vessels would also be permeable to fluid and solute. However, a functional role for VE-cadherin in the control of lymphatic vascular permeability has not yet been demonstrated. Recent findings by our group showed that lymphatic-specific deletion of VE-cadherin for 2 weeks caused severe lymph leakage and chylous ascites (Yang et al., 2019), suggesting that this adherens junction protein was critical for regulating lymphatic integrity during postnatal development. However, genetic deletion of VE-cadherin for 1 week did not cause any visible lymph leakage or chylous ascites. These findings are supported by an earlier study in which VE-cadherin was deleted from all endothelium but was not associated with hemorrhage or increased permeability of the skin or brain vasculatures (Frye et al., 2015). To clarify whether VE-cadherin regulates lymphatic vascular permeability, and to circumvent complications of gene inactivation, we acutely treated collecting lymphatic vessels with a function blocking antibody (BV13) to physically disrupt VE-cadherin binding (Corada et al., 1999) before quantifying their permeability to a small (3 kDa dextran) and a large (BSA) molecular weight solute. We found that disruption of VE-cadherin binding in collecting lymphatic vessels significantly and dramatically increased P_s_, demonstrating a direct role for this key adherens junction protein in regulating lymphatic permeability. Immunostaining showed more diffuse VE-cadherin localization following BV13 treatment, consistent with previous studies (Corada et al., 1999; Baluk et al., 2007). Disruption of VE-cadherin binding did not discriminate between solutes of different sizes, causing a significant increase in P_s_ to both small and large solutes and both protein and sugars. These findings suggest that VE-cadherin is essential for maintaining the barrier function of collecting lymphatic vessels, and that its dysregulation will have severe consequences. We predict that massive loss of BSA from lymph into the interstitial space would disrupt the oncotic pressure gradient across the lymphatic wall. Lymph fluid would then follow BSA into the interstitial space rather than returning to the bloodstream, leading to tissue edema and reduced vascular volume.

### Comparison to Previous Reports of Venular Solute P_s_

In our original report, we accepted the null hypothesis that the permeability to albumin did not differ between collecting lymphatic vessels and venules. Here, we now show that BSA uses a vesicular transport pathway and thus may be regulated differently from other solutes. To gain a more complete view of how collecting lymphatic permeability compares to the permeability of veins, we can compare our values here to previous reports of venular permeability to the same solutes. The mean permeability of frog venules to NaFl was 344 ± 145 × 10^–7^ cm s^–1^ (Fu et al., 1998), which agrees well with the mean permeability of rat venules to NaFl of 380 ± 30 × 10^–7^ cm s^–1^ (Montermini et al., 2002). Both of these values are approximately 1.8–2-fold higher than our mean P_s_ of collecting lymphatics to NaFl at a similar pressure of 5 cmH_2_O of 192 ± 158 × 10^–7^ cm s^–1^. The mean venular P_s_ to 3 kDa dextran was reported to be 48 ± 18 × 10^–7^ cm s^–1^ (Curry et al., 1983), which is 1.4-fold higher than our collecting lymphatic P_s_ of 34 ± 25 × 10^–7^ cm s^–1^. Finally, the mean venular P_s_ to α-lactalbumin was reported to be 21 × 10^–7^ cm s^–1^ at a pressure of 3 cmH_2_O (Huxley et al., 1987), which is 2.5-fold higher than our value of 7.8 ± 9.8 × 10^–7^ cm s^–1^ for collecting lymphatic vessels. Taken together, the solute permeability of collecting lymphatic vessels is consistently lower than that of venules, although paired experiments need to be performed in a future study to confirm whether this is a significant difference.

### Estimation of Lymphatic Hydraulic Conductivity From P_s_

While we report the solute permeability of collecting lymphatic vessels here, to our knowledge, no direct measurements of hydraulic conductivity have been made in lymphatic vessels. Hydraulic conductivity (L_*p*_) can be understood as the “permeability” of a vessel to water, albeit with units of cm s^–1^ cmH_2_O^–1^, which represents permeability (cm s^–1^) normalized to hydrostatic pressure (cmH_2_O). While water has a small molecular weight of only 18 g mol^–1^, we realized that we approached this size with the smallest solute used here, NaFl at 376 g mol^–1^. Since the NaFl P_s_ was plotted against pressure in Figure 2D, we can perform linear regression to obtain the slope of this relationship, resulting in a measurement that establishes a lower boundary for the hydraulic conductivity of collecting lymphatic vessels. The slope of that graph is equal to 17.3 × 10^–7^ cm s^–1^ cmH_2_O^–1^. Therefore, we expect that the hydraulic conductivity of collecting lymphatic vessels will be even higher than this value. Interestingly, this means that collecting lymphatic vessels have an approximately 10–17-fold higher L_*p*_ when compared to venules that have an L_*p*_ of approximately 1–2 × 10^–7^ cm s^–1^.cmH_2_O^–1^ (Kendall and Michel, 1995; Curry et al., 2003).

## Future Directions

In this study, we chose to measure permeability in collecting lymphatic vessels isolated from the mesentery. Previous research has shown that the permeability to BSA did not differ between collecting lymphatics vessels isolated from the mesentery and the popliteal region (Ivanov et al., 2016). Nevertheless, it is still possible that the permeability of collecting lymphatic vessels to other solutes, such as NaFl and 3 kDa dextran, could be tissue specific. Future studies should compare the permeability of lymphatic vessels in different tissue beds to determine whether permeability is tissue-specific.

Previous research has shown that the permeability of blood microvessels can be affected by the charge of the solute (Adamson et al., 1988; Curry et al., 1989). Microvessel permeability to the anionic solute, α-lactalbumin, is reduced in the presence of other anionic solutes such as BSA by exclusion from the vessel wall (Huxley et al., 1993). Where possible, we have utilized neutral solutes to eliminate any differences in permeability being due to differences in charge. However, we also included in our study α-lactalbumin and BSA, which both carry negative charges, since these solutes have historically been used in previous studies of permeability in blood vessels (Huxley et al., 1987) and lymphatic vessels (Scallan and Huxley, 2010; Scallan et al., 2015), and we wanted to make comparisons to previously published reports. Future studies may be carried out to determine how solute charge modulates lymphatic vessel permeability.

## Conclusion

In conclusion, our data reveal that lymphatic endothelial permeability is inversely related to solute size and directly related to pressure, indicating that solutes are preferentially moving via size-dependent pathways such as intercellular junctions. We showed that the transport of multiple solutes is regulated by VE-cadherin at the intercellular junctions, while BSA is selectively transported transcellularly via vesicles. These findings suggest that VE-cadherin is required for ensuring barrier function in the lymphatic vasculature, and that its disruption would likely lead to the extravasation of most solutes into the interstitium, which would pull lymph fluid into the tissues to cause edema. Intriguingly, tissue edema and reduced vascular volume occur in sepsis, so future studies should determine whether increased lymphatic vascular permeability plays a role in the pathogenesis of sepsis.

## Data Availability Statement

The original contributions presented in the study are included in the article/supplementary material, further inquiries can be directed to the corresponding author.

## Ethics Statement

The animal study was reviewed and approved by Institutional Animal Care and Use Committee of the University of South Florida.

## Author Contributions

Both authors conceived, designed, and performed experiments, acquired and analyzed data, interpreted the results, drafted and revised the manuscript, approved the final version of the manuscript, and agreed to be accountable for all aspects of the work. All individuals listed as author qualify for authorship.

## Conflict of Interest

The authors declare that the research was conducted in the absence of any commercial or financial relationships that could be construed as a potential conflict of interest.

## Publisher’s Note

All claims expressed in this article are solely those of the authors and do not necessarily represent those of their affiliated organizations, or those of the publisher, the editors and the reviewers. Any product that may be evaluated in this article, or claim that may be made by its manufacturer, is not guaranteed or endorsed by the publisher.

## References

[B1] Adamson R. H. Huxley V. H. Curry F. E. (1988). Single capillary permeability to proteins having similar size but different charge. *Am. J. Physiol.* 254 H304–H312. 3257846 10.1152/ajpheart.1988.254.2.H304

[B2] Avraham T. Zampell J. C. Yan A. Elhadad S. Weitman E. S. Rockson S. G. (2013). Th2 differentiation is necessary for soft tissue fibrosis and lymphatic dysfunction resulting from lymphedema. *FASEB J.* 27 1114–1126. 10.1096/fj.12-222695 23193171 PMC3574290

[B3] Baluk P. Fuxe J. Hashizume H. Romano T. Lashnits E. Butz S. (2007). Functionally specialized junctions between endothelial cells of lymphatic vessels. *J. Exp. Med.* 204 2349–2362. 10.1084/jem.20062596 17846148 PMC2118470

[B4] Bouta E. M. Wood R. W. Brown E. B. Rahimi H. Ritchlin C. T. Schwarz E. M. (2014). In vivo quantification of lymph viscosity and pressure in lymphatic vessels and draining lymph nodes of arthritic joints in mice. *J. Physiol.* 592 1213–1223. 10.1113/jphysiol.2013.266700 24421350 PMC3961082

[B5] Brouillard P. Boon L. Vikkula M. (2014). Genetics of lymphatic anomalies. *J. Clin. Invest.* 124 898–904. 24590274 10.1172/JCI71614 PMC3938256

[B6] Corada M. Mariotti M. Thurston G. Smith K. Kunkel R. Brockhaus M. (1999). Vascular endothelial-cadherin is an important determinant of microvascular integrity in vivo. *Proc. Natl. Acad. Sci. U. S. A.* 96 9815–9820. 10.1073/pnas.96.17.9815 10449777 PMC22293

[B7] Curry F. E. Huxley V. H. Adamson R. H. (1983). Permeability of single capillaries to intermediate-sized colored solutes. *Am. J. Physiol.* 245 H495–H505. 6604463 10.1152/ajpheart.1983.245.3.H495

[B8] Curry F. E. Rutledge J. C. Lenz J. F. (1989). Modulation of microvessel wall charge by plasma glycoprotein orosomucoid. *Am. J. Physiol.* 257 H1354–H1359. 2589490 10.1152/ajpheart.1989.257.5.H1354

[B9] Curry F. E. Zeng M. Adamson R. H. (2003). Thrombin increases permeability only in venules exposed to inflammatory conditions. *Am. J. Physiol. Heart Circ. Physiol.* 285 H2446–H2453. 12893636 10.1152/ajpheart.00262.2003

[B10] Dejana E. Orsenigo F. Lampugnani M. G. (2008). The role of adherens junctions and VE-cadherin in the control of vascular permeability. *J. Cell Sci.* 121 2115–2122. 10.1242/jcs.017897 18565824

[B11] Dejana E. Vestweber D. (2013). The role of VE-cadherin in vascular morphogenesis and permeability control. *Prog. Mol. Biol. Transl. Sci.* 116 119–144. 10.1016/b978-0-12-394311-8.00006-6 23481193

[B12] Fonseca D. M. Hand T. W. Han S. J. Gerner M. Y. Glatman Zaretsky A. Byrd A. L. (2015). Microbiota-Dependent Sequelae of Acute Infection Compromise Tissue-Specific Immunity. *Cell* 163 354–366. 10.1016/j.cell.2015.08.030 26451485 PMC4826740

[B13] Frye M. Dierkes M. Kuppers V. Vockel M. Tomm J. Zeuschner D. (2015). Interfering with VE-PTP stabilizes endothelial junctions in vivo via Tie-2 in the absence of VE-cadherin. *J. Exp. Med.* 212 2267–2287. 10.1084/jem.20150718 26642851 PMC4689167

[B14] Fu B. M. Adamson R. H. Curry F. E. (1998). Test of a two-pathway model for small-solute exchange across the capillary wall. *Am. J. Physiol.* 274 H2062–H2073. 9841533 10.1152/ajpheart.1998.274.6.H2062

[B15] Ghitescu L. Fixman A. Simionescu M. Simionescu N. (1986). Specific binding sites for albumin restricted to plasmalemmal vesicles of continuous capillary endothelium: receptor-mediated transcytosis. *J. Cell Biol.* 102 1304–1311. 10.1083/jcb.102.4.1304 3007533 PMC2114181

[B16] Grimsley-Myers C. M. Isaacson R. H. Cadwell C. M. Campos J. Hernandes M. S. Myers K. R. (2020). VE-cadherin endocytosis controls vascular integrity and patterning during development. *J. Cell Biol.* 219:e201909081. 10.1083/jcb.201909081 PMC7199849 32232465

[B17] Harvey N. L. Srinivasan R. S. Dillard M. E. Johnson N. C. Witte M. H. Boyd K. (2005). Lymphatic vascular defects promoted by Prox1 haploinsufficiency cause adult-onset obesity. *Nat. Genet.* 37 1072–1081. 10.1038/ng1642 16170315

[B18] Huxley V. H. Curry F. E. Adamson R. H. (1987). Quantitative fluorescence microscopy on single capillaries: alpha-lactalbumin transport. *Am. J. Physiol.* 252 H188–H197. 3492924 10.1152/ajpheart.1987.252.1.H188

[B19] Huxley V. H. Curry F. E. Powers M. R. Thipakorn B. (1993). Differential action of plasma and albumin on transcapillary exchange of anionic solute. *Am. J. Physiol.* 264 H1428–H1437. 8498557 10.1152/ajpheart.1993.264.5.H1428

[B20] Ivanov S. Scallan J. P. Kim K. W. Werth K. Johnson M. W. Saunders B. T. (2016). CCR7 and IRF4-dependent dendritic cells regulate lymphatic collecting vessel permeability. *J. Clin. Invest.* 126 1581–1591. 10.1172/jci84518 26999610 PMC4811132

[B21] Kataru R. P. Baik J. E. Park H. J. Wiser I. Rehal S. Shin J. Y. (2019). Regulation of Immune Function by the Lymphatic System in Lymphedema. *Front. Immunol.* 10:470. 10.3389/fimmu.2019.00470 30936872 PMC6431610

[B22] Kendall S. Michel C. C. (1995). The measurement of permeability in single rat venules using the red cell microperfusion technique. *Exp. Physiol.* 80 359–372. 10.1113/expphysiol.1995.sp003853 7640005

[B23] Kuan E. L. Ivanov S. Bridenbaugh E. A. Victora G. Wang W. Childs E. W. (2015). Collecting lymphatic vessel permeability facilitates adipose tissue inflammation and distribution of antigen to lymph node-homing adipose tissue dendritic cells. *J. Immunol.* 194 5200–5210. 10.4049/jimmunol.1500221 25917096 PMC4433841

[B24] Lampugnani M. G. Resnati M. Raiteri M. Pigott R. Pisacane A. Houen G. (1992). A novel endothelial-specific membrane protein is a marker of cell-cell contacts. *J. Cell Biol.* 118 1511–1522. 10.1083/jcb.118.6.1511 1522121 PMC2289607

[B25] Lapinski P. E. Kwon S. Lubeck B. A. Wilkinson J. E. Srinivasan R. S. Sevick-Muraca E. (2012). RASA1 maintains the lymphatic vasculature in a quiescent functional state in mice. *J. Clin. Invest.* 122 733–747. 10.1172/jci46116 22232212 PMC3266774

[B26] Lim H. Y. Rutkowski J. M. Helft J. Reddy S. T. Swartz M. A. Randolph G. J. (2009). Hypercholesterolemic mice exhibit lymphatic vessel dysfunction and degeneration. *Am. J. Pathol.* 175 1328–1337. 10.2353/ajpath.2009.080963 19679879 PMC2731150

[B27] Mayerson H. S. (1963). On Lymph and Lymphatics. *Circulation* 28 839–842. 14079186 10.1161/01.cir.28.5.839

[B28] Milici A. J. Watrous N. E. Stukenbrok H. Palade G. E. (1987). Transcytosis of albumin in capillary endothelium. *J. Cell Biol.* 105 2603–2612. 10.1083/jcb.105.6.2603 3320050 PMC2114713

[B29] Montermini D. Winlove C. P. Michel C. (2002). Effects of perfusion rate on permeability of frog and rat mesenteric microvessels to sodium fluorescein. *J. Physiol.* 543 959–975. 10.1113/jphysiol.2002.023010 12231651 PMC2290533

[B30] Ogata F. Fujiu K. Matsumoto S. Nakayama Y. Shibata M. Oike Y. (2016). Excess Lymphangiogenesis Cooperatively Induced by Macrophages and CD4(+) T Cells Drives the Pathogenesis of Lymphedema. *J. Invest. Dermatol.* 136 706–714. 10.1016/j.jid.2015.12.001 27015456

[B31] Ono N. Mizuno R. Ohhashi T. (2005). Effective permeability of hydrophilic substances through walls of lymph vessels: roles of endothelial barrier. *Am. J. Physiol. Heart Circ. Physiol.* 289 H1676–H1682. 15964919 10.1152/ajpheart.01084.2004

[B32] Orsenigo F. Giampietro C. Ferrari A. Corada M. Galaup A. Sigismund S. (2012). Phosphorylation of VE-cadherin is modulated by haemodynamic forces and contributes to the regulation of vascular permeability in vivo. *Nat. Commun.* 3:1208. 10.1038/ncomms2199 PMC3514492 23169049

[B33] Ozeki M. Fukao T. (2019). Generalized Lymphatic Anomaly and Gorham-Stout Disease: overview and Recent Insights. *Adv. Wound Care* 8 230–245. 10.1089/wound.2018.0850 31236308 PMC6589502

[B34] Razani B. Engelman J. A. Wang X. B. Schubert W. Zhang X. L. Marks C. B. (2001). Caveolin-1 null mice are viable but show evidence of hyperproliferative and vascular abnormalities. *J. Biol. Chem.* 276 38121–38138. 10.1074/jbc.m105408200 11457855

[B35] Sabine A. Bovay E. Demir C. S. Kimura W. Jaquet M. Agalarov Y. (2015). FOXC2 and fluid shear stress stabilize postnatal lymphatic vasculature. *J. Clin. Invest.* 125 3861–3877. 10.1172/jci80454 26389677 PMC4607114

[B36] Sawane M. Kajiya K. Kidoya H. Takagi M. Muramatsu F. Takakura N. (2013). Apelin inhibits diet-induced obesity by enhancing lymphatic and blood vessel integrity. *Diabetes* 62 1970–1980. 10.2337/db12-0604 23378608 PMC3661640

[B37] Scallan J. P. Davis M. J. Huxley V. H. (2013). Permeability and contractile responses of collecting lymphatic vessels elicited by atrial and brain natriuretic peptides. *J. Physiol.* 591 5071–5081. 10.1113/jphysiol.2013.260042 23897233 PMC3810810

[B38] Scallan J. P. Hill M. A. Davis M. J. (2015). Lymphatic vascular integrity is disrupted in type 2 diabetes due to impaired nitric oxide signalling. *Cardiovasc. Res.* 107 89–97. 10.1093/cvr/cvv117 25852084 PMC4490202

[B39] Scallan J. P. Huxley V. H. (2010). In vivo determination of collecting lymphatic vessel permeability to albumin: a role for lymphatics in exchange. *J. Physiol.* 588 243–254. 10.1113/jphysiol.2009.179622 19917564 PMC2821562

[B40] Schnitzer J. E. (1992). gp60 is an albumin-binding glycoprotein expressed by continuous endothelium involved in albumin transcytosis. *Am. J. Physiol.* 262 H246–H254. 1733316 10.1152/ajpheart.1992.262.1.H246

[B41] Schubert W. Frank P. G. Razani B. Park D. S. Chow C. W. Lisanti M. P. (2001). Caveolae-deficient endothelial cells show defects in the uptake and transport of albumin in vivo. *J. Biol. Chem.* 276 48619–48622. 10.1074/jbc.c100613200 11689550

[B42] Semina E. V. Rubina K. A. Sysoeva V. Y. Rutkevich P. N. Kashirina N. M. Tkachuk V. A. (2014). Novel mechanisms regulating endothelial permeability via T-cadherin-dependent VE-cadherin phosphorylationand clathrin-mediated endocytosis. *Mol. Cell Biochem.* 387 39–53. 10.1007/s11010-013-1867-4 24136461 PMC3904039

[B43] Stan R. V. (2006). Endocytosis pathways in the endothelium: how many?. *Am. J. Physiol.* 209 L806–L808. 10.1152/ajplung.00533.2005 16603594

[B44] Tiruppathi C. Finnegan A. Malik A. B. (1996). Isolation and characterization of a cell surface albumin-binding protein from vascular endothelial cells. *Proc. Natl. Acad. Sci. U. S. A.* 93 250–254. 10.1073/pnas.93.1.250 8552615 PMC40216

[B45] Triacca V. Guc E. Kilarski W. W. Pisano M. Swartz M. A. (2017). Transcellular Pathways in Lymphatic Endothelial Cells Regulate Changes in Solute Transport by Fluid Stress. *Circ. Res.* 120 1440–1452. 10.1161/circresaha.116.309828 28130294

[B46] Vogel S. M. Minshall R. D. Pilipovic M. Tiruppathi C. Malik A. B. (2001). Albumin uptake and transcytosis in endothelial cells in vivo induced by albumin-binding protein. *Am. J. Physiol. Lung Cell. Mol. Physiol.* 281 L1512–L1522. 11704548 10.1152/ajplung.2001.281.6.L1512

[B47] Yang Y. Cha B. Motawe Z. Y. Srinivasan R. S. Scallan J. P. (2019). VE-Cadherin Is Required for Lymphatic Valve Formation and Maintenance. *Cell Rep.* 28 2397–2412.e4. 31461654 10.1016/j.celrep.2019.07.072 PMC6743082

[B48] Zhang F. Zarkada G. Yi S. Eichmann A. (2020). Lymphatic Endothelial Cell Junctions: molecular Regulation in Physiology and Diseases. *Front. Physiol.* 11:509. 10.3389/fphys.2020.00509 32547411 PMC7274196

[B49] Zweifach B. W. Prather J. W. (1975). Micromanipulation of pressure in terminal lymphatics in the mesentery. *Am. J. Physiol.* 228 1326–1335. 1130536 10.1152/ajplegacy.1975.228.5.1326

